# Optimization of Fluorescent Labeling for* In Vivo* Nanoimaging of Sarcomeres in the Mouse Heart

**DOI:** 10.1155/2018/4349170

**Published:** 2018-08-23

**Authors:** Fuyu Kobirumaki-Shimozawa, Togo Shimozawa, Kotaro Oyama, Yasuharu Kushida, Takako Terui, Shin'ichi Ishiwata, Norio Fukuda

**Affiliations:** ^1^Department of Cell Physiology, The Jikei University School of Medicine, 3-25-8 Nishi-shinbashi, Minato-ku, Tokyo 105-8461, Japan; ^2^Technical Division, School of Science, The University of Tokyo, 7-3-1 Hongo, Bunkyo-ku, Tokyo 113-0033, Japan; ^3^Department of Anesthesiology, The Jikei University School of Medicine, 3-25-8 Nishi-shinbashi, Minato-ku, Tokyo 105-8461, Japan; ^4^Department of Physics, Faculty of Science and Engineering, Waseda University, 3-14-9 Okubo, Shinjuku-ku, Tokyo 169-0072, Japan

## Abstract

The present study was conducted to systematically investigate the optimal viral titer as well as the volume of the adenovirus vector (ADV) that expresses *α*-actinin-AcGFP in the Z-disks of myocytes in the left ventricle (LV) of mice. An injection of 10 *μ*L ADV at viral titers of 2 to 4 × 10^11^ viral particles per mL (VP/mL) into the LV epicardial surface consistently expressed *α*-actinin-AcGFP in myocytes* in vivo*, with the fraction of AcGFP-expressing myocytes at ~10%. Our analysis revealed that SL was ~1.90-2.15 *μ*m upon heart arrest via deep anesthesia. Likewise, we developed a novel fluorescence labeling method of the T-tubular system by treating the LV surface with CellMask Orange (CellMask). We found that the T-tubular distance was ~2.10-2.25 *μ*m, similar to SL, in the healthy heart* in vivo*. Therefore, the present high-precision visualization method for the Z-disks or the T-tubules is beneficial to unveiling the mechanisms of myocyte contraction in health and disease* in vivo*.

## 1. Introduction

Recent advances in microscopic optics have enabled measurements of sarcomere length (SL) at high spatial and temporal resolution in living cultured or isolated cardiac cells (e.g., [1, 2]). Likewise, efforts have been made to visualize and analyze the dynamics of sarcomeres as well as cardiomyocytes in the heart* in vivo*. To our knowledge, Lee et al. (2012) were the first to image cardiomyocytes (and coronary vessels)* in vivo*, i.e., in anesthetized mice [3]. In 2014, Aguirre and colleagues captured images of the fluorescence-labeled T-tubules in the mouse heart, via two-photon microscopy following reconstruction of the original images [4]. However, the confocal system combined with a two-photon microscope has substantial limitations in the imaging of consistently working cardiac muscle* in vivo*, because the image acquisition time is extremely long compared with the physiologically relevant heartbeat frequencies of rodents (as discussed in [5]). In order to solve the shortcomings of these previous studies, we recently developed a new microscopic system for high-precision measurements of SL in mice via expression of *α*-actinin-AcGFP in the left ventricle (LV) [6].

In the present study, first, we systematically quantified the optimal viral titer and volume of the adenovirus vector (ADV) for effective expression of *α*-actinin-AcGFP in myocytes of the mouse heart to achieve high-precision SL analysis* in vivo*. Second, we developed a novel method to fluorescently label the T-tubular system by treating the epicardial surface with the plasma membrane stain CellMask Orange (CellMask), which allowed for visualization of the T-tubular system in myocytes connected in series in the heart* in vivo*.

## 2. Materials and Methods

This study was performed in accordance with the Guidelines on Animal Experimentation of The Jikei University School of Medicine. All animal experiments, approved by the Animal Care Committee of The Jikei University School of Medicine and the Recombinant Gene Research Safety Committee of The Jikei University School of Medicine, were reported in accordance with the ARRIVE guidelines.

### 2.1. Vector Construction

ADV was constructed based on our previous study [6]. In brief, recombinant adenoviruses encoding mouse *α*-actinin-3-AcGFP [Genbank/EMBL/DDBJ accession no. NM_013456] (Ad-actinin-AcGFP) were constructed by using the AdMax adenovirus vector creation kit (Microbix Biosystems Inc., Toronto, ON, Canada). The 6th Ad-actinin-AcGFP was used.

### 2.2. Purification of Adenoviruses

ADV was purified by using the Vivapure AdenoPACK 20 purification kit (Sartorius AG, Weender Landstr, Göettingen, Germany) [6]. The ADVs were concentrated by ultrafiltration to yield 2 × 10^11^–2 × 10^12^ viral particles per mL (VP/mL) in PBS (-). ADVs were stocked at –80°C for up to 2 months.

### 2.3. ADV Injection in the Heart* In Vivo*

ADV was injected into the heart of male BALB/c mice (*n *= 13, 3–8 weeks of age; Japan SLC, Shizuoka, Japan) anesthetized with ~2% isoflurane, as in our previous study [6]. In brief, left thoracotomy was performed in order to visualize the anterior surface of the LV under ventilation. The animal was warmed at 38°C. PBS (-) containing ADV at various viral titers was injected into the epicardial surface of the central region of the LV (10 or 30 *μ*L in ~3 × 3 mm^2^, ~10 spots) by using a 1 mL syringe pump with a 32G needle. Two days after chest closure, the mouse was anesthetized again with ~2% isoflurane and ventilated, and the anterior thoracic wall was removed by cutting the ribs, muscles, and intercostal arteries with the electric scalpel for* in vivo* cardiac sarcomere imaging.

### 2.4. CellMask Treatment

The plasma membrane stain CellMask Orange (CellMask; Thermo Fisher Scientific Inc., Waltham, MA, USA) was dissolved in PBS (-) (termed “CellMask solution”) and administered in anesthetized open-chest male BALB/c mice (*n* =3, 9–18 weeks of age; Japan SLC, Shizuoka, Japan) warmed at 38°C in three different ways. First, 200 *μ*L of the CellMask solution at a concentration of 5 *μ*g/mL was injected into the LV cavity via a 27G needle (in ~10 sec) while the aorta was clamped distal to the takeoff of the left subclavian artery using a microsyringe [7]. Following the CellMask injection, the aortic clamp was slowly released (in ~5 sec) to avoid occurrence of arrhythmias due to ischemia/reperfusion. Second, 10 *μ*L of the CellMask solution at a concentration of 0.5 *μ*g/mL was injected (in ~5 sec) into the epicardial surface of the central part of the LV. Third, a small piece of gauze was placed on the LV surface, and the CellMask solution at a concentration of 0.1 *μ*g/mL was gently dropped onto it for 5 min using a pipette. The three methods were performed in separate animals.

### 2.5. Microscopic System

The details of the microscopic system for* in vivo* nanoimaging have been described in our previous studies [5, 6]. In brief, an upright microscope (BX-51WI, Olympus Co., Tokyo, Japan) combined with a Nipkow confocal scanner (CSU21, Yokogawa Electric Co., Tokyo, Japan) and an electron multiplying CCD (EMCCD) camera (iXonEM+, Andor Technology Ltd, Belfast, Northern Ireland) were used at a 512 × 512 (or 512 × 170) pixel resolution at an exposure time of 28 (or 9.8) ms. A water immersion lens, either 60× (LUMPLFLN 60XW, N/A 1.00, Olympus Co.), 40× (LUMPLFLN 40XW, N/A 0.80, Olympus Co.), or 20× (XLUMPLFLN 20XW, N/A 1.00, Olympus Co.), and also a 2× lens (XLFluor 2X/340, N/A 0.14, Olympus Co.) were used to visualize the LV surface.

AcGFP-expressing myocytes were excited by a 488 nm laser light (HPU50211-PFS, Furukawa Electric Co., Tokyo, Japan), and the resultant fluorescence signals (emission filter: BA510–550, Olympus Co., Tokyo, Japan) were detected. In the experiments with CellMask, the heart was excited at 532 nm (MiniGreen FCIM-100; Snake Creek Lasers, Friendsville, PA, USA), and the resultant fluorescence signals (emission filter: BA575IF, Olympus Co.) were detected. When excited at 532 nm, the wavelength range for the detection of the fluorescence of CellMask was >575 nm.

### 2.6. Analysis of SL or the T-Tubular Distance

As in our previous studies [6, 8], SL or the T-tubular distance was measured by analyzing the fluorescence plot profiles along the longitudinal axis of a myocyte (or myocytes) by using the ImageJ software (National Institutes of Health, Bethesda, MD, USA) [the region of interest (ROI) width, 5 pixels]. Briefly, the profiles were analyzed using the multipeak Gaussian fitting with a linear function of offset (*Y *= a*X* + b), based on the Levenberg-Marquardt algorithm, and SL or the T-tubular distance was calculated as the distance between the centers of two adjacent peaks (see [1] for details). To minimize the error, sarcomeres or the T-tubules showing insufficient fluorescence intensity (FI) were not used.

### 2.7. *In Vivo* Nanoimaging

Nanoimaging was performed as in our previous studies [5, 6]. Namely, the anesthetized open-chest mouse under ventilation was placed on a custom-made microscope stage (200 × 250 mm^2^), and the animal was warmed at 38°C throughout imaging. ECG lead III was recorded. Likewise, the left ventricular pressure (LVP) was recorded by a catheter (FTH-1211B-0018, Transonic Systems Inc., Ithaca, NY, USA) inserted from the apex of the heart. A coverslip (0.04–0.06 mm thickness; No. 000, Matsunami Glass Ind., Osaka, Japan) (diameter, 12 mm) was gently attached to the LV surface at two points (~2 mm apart) using glue (Aron Alpha, TOA GOSEI Co., Tokyo, Japan). The position of the coverslip was carefully controlled by a custom-made micromanipulator (Sigma Koki Co., Tokyo, Japan). For both AcGFP and CellMask, imaging was started upon heart arrest via deep anesthesia with ~5% isoflurane. Heart arrest was defined as no signal of either ECG or LVP. The ventilator was turned off during imaging.

We observed the first layer of myocytes under the mesothelial cells, and the fraction of AcGFP-expressing myocytes was defined as the ratio of the area of fluorescence labelled myocytes to that of the total area in the confocal microscopic field. The ratio values were averaged with the results of individual animals.

### 2.8. Statistical Analysis

Data are expressed as mean±SD. Significant differences were tested by Welch's *t*-test unless otherwise noted.

## 3. Results

### 3.1. Expression of AcGFP in Cardiomyocytes via ADV Injection* In Vivo*

**Figure 1:**
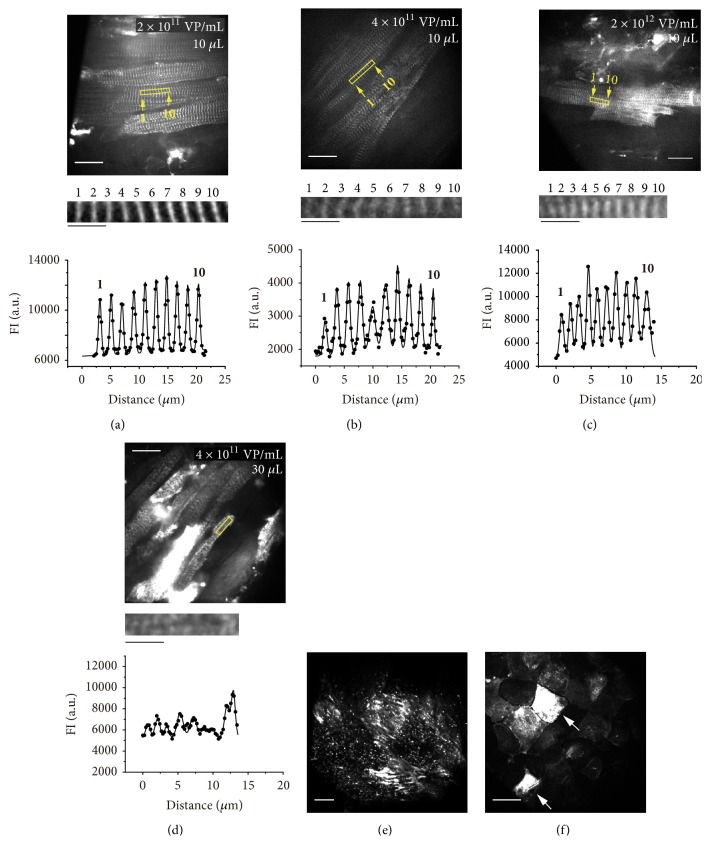
**Expression of** **α** **-actinin-AcGFP in cardiomyocytes in mice. (a) Top**, confocal image showing ventricular myocytes expressed with *α*-actinin-AcGFP by an ADV injection of 10 *μ*L at 2 × 10^11^ VP/mL. Values on ADV shown in the upper right corner.** Middle**: enlarged view of the region of interest (i.e., yellow outlined rectangle) in the confocal image in** Top. Bottom**: FI profile of the confocal image. Closed circles, plot profile of the image (raw data). Solid line, data analyzed by the multipeak Gaussian fitting. Numbers in the graph indicate the Z-disks in the confocal image. Average distances between FI peaks (i.e., SL), 1.92±0.08 *μ*m. Note that this method collectively derives all of the SL (or T-tubular distance; see**Figure 3(e)**) values along the region of interest.** (b) **Same as in** (a)** by an ADV injection of 10 *μ*L at 4 × 10^11^ VP/mL. Average length of 9 sarcomeres, 2.10±0.09 *μ*m.** (c)** Same as in** (a)** by an ADV injection of 10 *μ*L at 2 × 10^12^ VP/mL. Average length of 9 sarcomeres, 1.34±0.12 *μ*m. In** (a)**–**(c)**:** Top**, Bar = 20 *μ*m.** Middle**, Bar = 5 *μ*m. The Z-disks are numbered in confocal images, and “1” and “10” in FI profiles indicate fitted fluorescence signals for the corresponding Z-disks. Observed with a 60× lens (exposure time, 28 ms). See**Table 1** for summarized SL data.** (d) Top**: same as in** (a)** by an ADV injection of 30 *μ*L at 4 × 10^11^ VP/mL. Bar = 20 *μ*m.** Middle**: enlarged view of the region of interest (i.e., yellow outlined rectangle) in the confocal image in** Top**. Bar = 5 *μ*m. Length of 8 sarcomeres, 1.52±0.44 *μ*m. P<0.05 compared with the value in** (a)** or** (b)**. Observed with a 60× lens (exposure time, 28 ms). Note disordered striation patterns in myocytes.** (e)** Confocal image of the ADV-injected area in the LV surface in** (d)**. Bar = 500 *μ*m. Observed with a 2× lens (exposure time, 28 ms).** (f)** Confocal image of mesothelial cells [just above the first layer of cardiomyocytes, i.e., shown in** (d)**]. Note the clear fluorescence signal in some mesothelial cells (shown by arrows). Bar = 20 *μ*m. Observed with a 60× lens (exposure time, 28 ms).

First, we confirmed whether or not an ADV injection into the surface of the LV expresses AcGFP in the Z-disks of cardiomyocytes in mice. An ADV injection of 10 *μ*L at 2 × 10^11^ or 4 × 10^11^ VP/mL into the epicardial surface effectively expressed AcGFP in the Z-disks (**Figures 1(a) and 1(b)**), with fraction of the AcGFP-expressing myocytes at ~10% in the injected region (~3 × 3 mm^2^). SL was 1.91±0.23 and 2.15±0.19 *μ*m with 2 × 10^11^ and 4 × 10^11^ particles per mL (**Table 1**), respectively, both of which, measured* in vivo*, are consistent with the resting value of 1.97±0.20 *μ*m obtained in our previous study in the isolated mouse heart [6]. An increase in the viral titer to 2 × 10^12^ particles per mL increased the fraction of the AcGFP-expressing myocytes to ~20-30%; however, this caused marked shortening of sarcomeres (**Figure 1(c); Table 1**). Moreover, this titer of ADV increased the background fluorescence from the surrounding myocytes/tissues, thus reducing the resolution of the SL analysis.

Then, in order to increase the fraction of AcGFP-expressing myocytes (hence the ease of sarcomere imaging), we increased the volume of ADV to 30 *μ*L at a viral titer of 4 × 10^11^ VP/mL. Although the fraction was increased to ~20-30%, sarcomere striations were markedly disordered in >~50% of AcGFP-expressing myocytes (**Figure 1(d)**). Even within a local region in a myocyte where striations remained, SL was shortened (**Table 1**).**Figure 1(e)** shows an epi-illumination image of the surface of the ADV-injected region in the LV of the mouse in**Figure 1(d)**. Strong fluorescence signals were observed even via epi-illumination, indicating high fractions of myocytes as well as mesothelial cells (**Figure 1(f)**) that express AcGFP.

In summary, an ADV injection of 10 *μ*L at 2-4 × 10^11^ VP/mL into the LV epicardial surface is best suited for the analysis of SL in highest possible resolution images of AcGFP-expressing myocytes.

### 3.2. Effect of Epicardial Treatment with Collagenase on the Expression of *α*-Actinin-AcGFP in the Z-Disks

Collagenase is widely used in the preparation of single cardiomyocytes from the isolated perfused heart ([2] and references therein). Given, therefore, its effect in degrading collagen and, presumably, the ensuing enhancement of infiltration of ADV around cardiomyocytes, the collagenase treatment in the LV prior to the ADV injection is expected to increase the fraction of *α*-actinin-AcGFP-expressing cardiomyocytes.

In order to definitely express *α*-actinin-AcGFP, only in this experiment, we used a slightly higher viral titer; namely, we treated the epicardial surface of the LV with collagenase at a concentration of 0.25 mg/ml (30 *μ*L) for 1 min, and subsequently, ADV (10 *μ*L, 8 × 10^11^ VP/mL) was injected.

**Figure 2:**
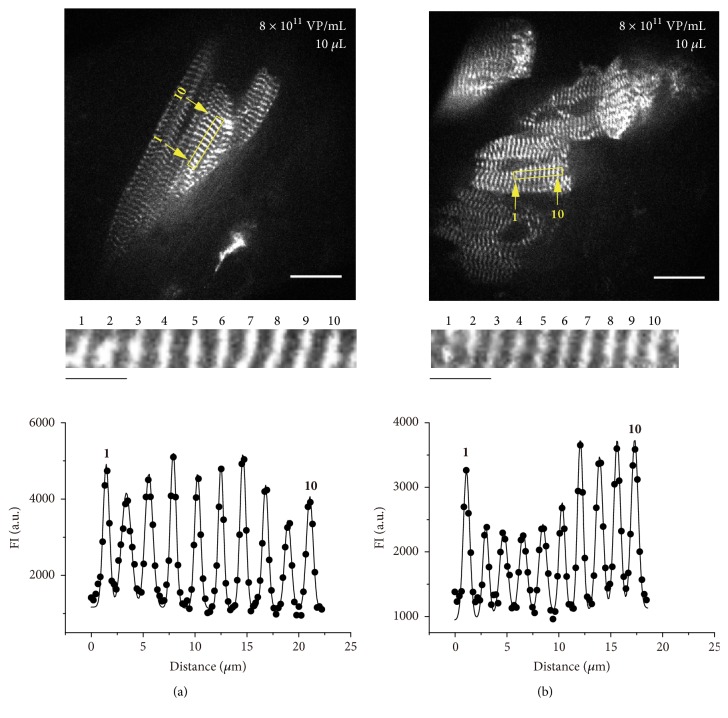
**Effect of collagenase treatment on the expression of** **α** **-actinin-AcGFP in cardiomyocytes* in vivo*. (a) Top**: confocal image showing uncontracted ventricular myocytes expressed with *α*-actinin-AcGFP by an ADV injection of 10 *μ*L at 8 × 10^11^ VP/mL at the center of the LV. The LV surface was pretreated with collagenase. Bar = 20 *μ*m**. Middle**: enlarged view of the region of interest (indicated by yellow-rectangular region) in the confocal image in** Top**. Bar = 5 *μ*m**. Bottom**: FI profile in the yellow-rectangular region in Top. Closed circles, plot profile of the image (raw data). Solid line, data analyzed by the multipeak Gaussian fitting. Length of 9 sarcomeres, 2.18±0.13 *μ*m.** (b)** Same as in** (a)** in contracted myocytes in the same animal. Note contraction of all myocytes shown in this image. Length of 9 sarcomeres, 1.80±0.08 *μ*m. P<0.001 compared with the values in** (a)**. In** (a)** and** (b)**: the Z-disks are numbered in confocal images, and “1” and “10” in FI profiles indicate fitted fluorescence signals for the corresponding Z-disks. Bar = 20 *μ*m. Observed with a 60× lens (exposure time, 28 ms).

While the AcGFP expression ratio was slightly increased to ~20-30%, approximately half of the myocytes were distorted and exhibited shortening (**Figures 2(a) and 2(b)**); SL was 2.18±0.13 and 1.80±0.08 *μ*m (P<0.001) in normal and distorted myocytes in the same heart, respectively.

It is therefore considered that the pretreatment with collagenase is not suitable for high-precision analysis of SL* in vivo*.

### 3.3. Visualization of the T-Tubules in Cardiomyocytes* In Vivo*

Previously we labeled the T-tubules of cardiomyocytes by perfusing the isolated heart via the aorta with Tyrode's solution containing CellMask (1 *μ*M), and determined the T-tubular distance at rest (i.e., ~2.0 *μ*m; [8]). In the present study, we investigated whether or not the heart's T-tubular system can be stained by using CellMask* in vivo*.

**Figure 3:**
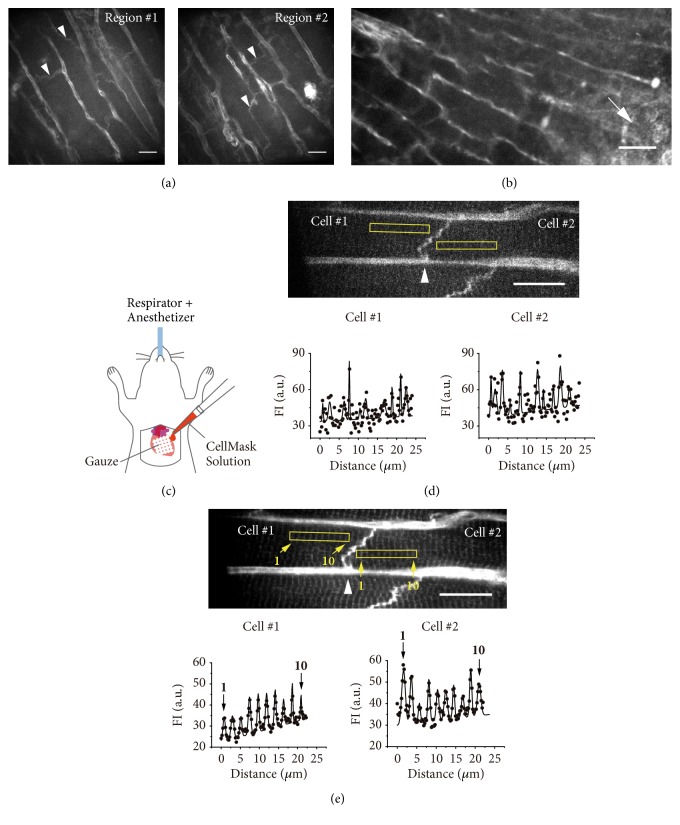
**Visualization of cardiomyocytes by CellMask in the LV* in vivo*. (a)** Confocal images showing myocytes as well as capillaries in two different regions (i.e., Regions #1 and #2) in the LV of a mouse. CellMask was injected into the LV chamber via the apex of the heart. Region #1 is at the center of the LV, and Region #2 is ~3 mm closer to the apex of the heart. Note marked FI in capillaries in both regions. Arrowheads indicate gap junctions. Bar = 20 *μ*m. In both images: observed with a 40× lens (exposure time, 28 ms).** (b)** Same as in** (a)** in the LV of a mouse, but CellMask (0.5 *μ*g/mL, 10 *μ*L) was directly injected into the epicardial surface. Arrow indicates the injected region showing fragmented cells. Gap junctions are not clearly observed by this method. Bar = 40 *μ*m. Observed with a 20× lens (exposure time, 28 ms).** (c)** Illustration showing the CellMask treatment on the LV epicardial surface. A small piece of gauze (~5 × 5 mm^2^) was gently placed on the LV surface and the CellMask solution (0.1 *μ*g/mL, ~1 mL) was dropped onto it for ~5 min using a pipette. The animal, anesthetized with 2% isoflurane, was ventilated during the CellMask treatment.** (d) Top**: confocal image showing myocytes in the instillation region in the LV of a mouse. Arrowhead indicates a gap junction. Bar = 20 *μ*m. Observed with a 60× lens (exposure time, 9.8 ms).** Bottom**: FI profile in the yellow-rectangular regions in Cells #1 and #2 in** Top**. T-tubular distance, 2.42±1.17 and 2.26±1.00 *μ*m in Cells #1 and #2, respectively.** (e) Top**: same as in** (d) Top** via superposition of 25 images [the same myocytes as in** (d) Top** are shown]. Arrowhead indicates a gap junction. Bar = 20 *μ*m.** Bottom**: FI profile in the yellow-rectangular regions in Cells #1 and #2 in** Top**. T-tubular distance, 2.24±0.05 and 2.10±0.23 *μ*m (P=0.15) in Cells #1 and #2, respectively. The T-tubules are numbered in the image, and “1” and “10” in FI profiles indicate fitted fluorescence signals for the corresponding T-tubules.

First, we injected the CellMask solution (i.e., 200 *μ*L) into the LV chamber from the apex of the heart with the aorta clamped (hence the heart was perfused with CellMask via coronary vessels) (**Figure 3(a)**). While capillaries and gap junctions were clearly stained by this method, as a result of the high background fluorescence, the T-tubules were not visualized (in either Region #1 or #2, ~3 mm apart in the same heart).

Second, we injected the CellMask solution (10 *μ*L) directly into the LV epicardial surface (**Figure 3(b)**). However, the CellMask injection caused necrosis in the injected region, and, as in the case above, the T-tubules were not visualized because of the high background fluorescence.

Finally, we placed a small piece of gauze on the LV surface and dropped the CellMask solution (1 mL) onto it for ~5 min (see illustration in**Figure 3(c)**). Although the FI of the sequential T-tubules varied along the myocyte, this method enabled visualization of the T-tubules in myocytes connected in series via a gap junction* in vivo*, at a short exposure time of 9.8 ms (**Figure 3(d)**). A superposition of 25 images within the same myocyte provided clearer fluorescence signals from the T-tubules, with the T-tubular distance of 2.24±0.05 and 2.11±0.23 *μ*m (P=0.15) in Cells #1 and #2, respectively. These values are similar to SL measured* in vivo* via AcGFP expression (see** Figures 1(a), 1(b), and 2(a)**).

## 4. Discussion

The findings of the present study are threefold. First, an ADV injection into the LV epicardial surface effectively expressed *α*-actinin-AcGFP in the Z-disks of myocytes in mice* in vivo*. Second, the pretreatment with collagenase increased the fraction of *α*-actinin-AcGFP-expressing myocytes; however, approximately half of the myocytes exhibited sarcomere shortening. Third, an epicardial treatment with CellMask enabled visualization of the T-tubules in sequentially connecting myocytes* in vivo*.

First, we systematically determined the optimal titer / volume of the ADV solution for effective expression of *α*-actinin-AcGFP in cardiomyocytes in mice. Namely, an injection of 10 *μ*L of 2-4 × 10^11^ particles per mL viral titer was best suited for the high-precision analysis of SL, although the fraction of AcGFP-expressing myocytes was relatively low at ~10% (**Figure 1**). An increase in the titer to 2 × 10^12^ VP/mL increased this fraction to ~20-30%, which, unfortunately, however, caused shortening of sarcomeres and an increase in the background fluorescence. It could therefore be concluded that, under the present optical setting with a Nipkow confocal unit, the fraction of AcGFP-expressing myocytes should be limited to ~10% for high-precision SL analysis in the heart* in vivo*. Likewise, an increase in the volume of the ADV solution to 30 *μ*L caused (1) disorganization of the sarcomere structure and (2) expression of AcGFP, not only in cardiomyocytes but also in mesothelial cells. Given the marked shortening of the remaining sarcomeres (i.e., 1.27±0.17 *μ*m;**Table 1**), we consider that the disordered internal structure is caused via the following mechanisms. First, excessive infection by ADV damages the sarcolemma and subsequently enhances the influx of Ca^2+^ into myocytes. Then, the subsequent release of Ca^2+^ from the sarcoplasmic reticulum contracts sarcomeres and concomitantly activates Ca^2+^-activated proteases (calpains) inside cardiomyocytes. Calpains break down the peptide bonds in sarcomeric structural proteins, especially the PEVK domain of titin in the I-band, resulting in the disorganization of sarcomeres via repeated contractions* in vivo* (e.g., [9]).

It is worthwhile to note that the orientation of a myocyte in the heart relative to the confocal image plane may result in an overestimation of SL. However, taking into consideration the careful analysis by the group of Bub [10, 11] combined with our SL analysis based on the Gaussian fitting, the overestimation is ~2% in the present study, because the length of in-focus images in the image plane (i.e., *L*_min_ in [10, 11]) was longer than ~70 *μ*m (e.g.,** Figures 1(a) and 1(b)**). It can therefore be considered that the influence of the myocyte angle on SL is practically negligible in the present experimental setting.

While the epicardial treatment with collagenase increased the fraction of *α*-actinin-AcGFP-expressing cardiomyocytes (to ~20-30%), it resulted in the shortening of sarcomeres in ~50% of the AcGFP-expressing myocytes. This may be due to a relatively high viral titer of ADV (i.e., 8 × 10^11^ VP/mL). It is, however, more likely that collagenase treatment increased the accessibility of ADV to myocytes, resulting in an increase in the influx of Ca^2+^ into the cells, based on the mechanism described above. It is well established that, in the heart, intracellular Ca^2+^ overload in myocytes results in the occurrence of arrhythmias due to depolarization of cellular membranes via influx of Na^+^ by means of the Na^+^-Ca^2+^ exchanger ([5] and references therein). Therefore, although the collagenase treatment (**Figure 2**) (or a large volume / a high viral particles of ADV) increases the expression fraction of AcGFP-expressing cardiomyocytes (in other words, enhances the ease of finding of fluorescently labeled cells), Ca^2+^-overloaded myocytes are likely to affect the heart's conductance system, thereby affecting the precision of the SL analysis.

We found in the present study that CellMask was effective for the* in vivo* measurement of the T-tubular distance, via treatment on the LV epicardial surface. It is generally considered that in healthy adult cardiomyocytes, the T-tubules and the Z-disks are closely associated and run in parallel, and, therefore, SL and the T-tubular distance are similar [12]. The group of Bub developed a method allowing for the measurement of the T-tubular distance in the isolated heart of the rat by perfusing the heart with the voltage sensitive dye di-4-ANNEPS (which effectively stains the myocardial membrane system) [10, 11]. However, here the perfusion of the heart with a fluorescence reagent (CellMask in the present study; cf.**Figure 3(a)**) predominantly stained capillaries, rather than myocytes, and, therefore, the T-tubular system was not sufficiently stained for high-precision measurement of the T-tubular distance at an exposure time required for cardiac nanoimaging in mice (see [13] for heart rates in mice). Likewise, an epicardial injection of CellMask caused necrosis in the injected region and increased the background fluorescence, and hence it proved unsuitable for the T-tubular distance analysis (**Figure 3(b))**.

Based on the results of** Figures 3(a) and 3(b)**, we developed a novel method to effectively stain the cardiomyocyte membranes as well as gap junctions* in vivo* with no appreciable damage to myocytes (**Figure 3(c)**), although the fluorescence signals were weaker than with AcGFP. Even a short exposure time of 9.8 ms allowed for visualization of the T-tubules in sequentially connecting myocytes, which cannot be achieved by an ADV injection, because the position of the intercalated discs between myocytes is unclear via *α*-actinin-AcGFP expression (cf.** Figures 1 and 2**). Here it may be pointed out that difference in the T-tubule distance values is due to low resolution coupled with a short exposure time, i.e., within an error. A superposition of the images in the same regions derived the T-tubular distance value as ~2.20 *μ*m in both myocytes (see**Figure 3** legend). It should be noted that SL and the T-tubular distance are similar (**Figures 1(a), 1(b), 2(a), and 3(e)**), close to the resting SL at the LV center in the isolated heart from our previous study [6]. Likewise, these values are in good agreement with those obtained by others [14] in epicardial myocytes in the LV of Langendorff perfused isolated mouse heart. Therefore, the present finding may strengthen the rationale for the use of the T-tubular distance as SL with membrane-staining reagents such as CellMask ([6, 8] and the present study) or di-4-ANNEPS [10, 11] in the isolated heart or the heart* in vivo*. However, di-4-ANNEPS reportedly slows the heart's conduction velocity and possibly affects subsequent mechanical properties [15]. Therefore, CellMask may be better suited for the analysis of SL dynamics with little or no influence on the heart's excitation-contraction coupling* in vivo*.

## 5. Conclusions

We demonstrated that in order to achieve high-precision SL analysis in cardiomyocytes* in vivo*, the fraction of AcGFP-expressing myocytes must be maintained low, i.e., at ~10%. Likewise, we developed a novel method by using CellMask to measure the T-tubular distance in sequentially connecting myocytes in the LV* in vivo*. Future studies are warranted to systematically investigate whether or not SL and the T-tubular distance are correlated and how these two parameters vary in accordance with the heart's contractile state in various locations of the heart* in vivo* in health and disease.

## Figures and Tables

**Table 1:** **Summary of the SL values via expression of *α*-actinin-AcGFP at various viral loads.** See **Figure 1** for details. ^*∗*^*P* < 0.001 compared with the value at 2×10^11^ VP/mL (10 *μ*L). ^†^*P* < 0.001 compared with the value at 4×10^11^ VP/mL (10 *μ*L). ‡ means *P* < 0.001 compared with the value at 2×10^12^ VP/mL (10 *μ*L). Significant differences were tested by the Steel-Dwass test.

Titer (VP/mL)	Volume (*μ*L)	SL (*μ*m)	Number of myocytes	Number of animals	Number of sarcomeres
2×10^11^	10	1.91 ± 0.23	38	3	1626
4×10^11^	10	2.15 ± 0.19^*∗*^	4	3	177
2×10^12^	10	1.42 ± 0.24^*∗*,†^	18	4	697
4×10^11^	30	1.27 ± 0.17^*∗*,†,‡^	8	3	347

## Data Availability

The data used to support the findings of this study are available from the corresponding authors upon request.

## Abbreviations

ADV: Adenovirus vector
CellMask: CellMask Orange
FI: Fluorescence intensity
LV: Left ventricle
SL: Sarcomere length.
